# Case report: Intravascular large B cell lymphoma mimicking acute demyelinating encephalomyelitis after SARS-CoV-2 reinfection: diagnostic value of advanced MRI techniques and the literature review with the assistance of ChatGPT

**DOI:** 10.3389/fimmu.2024.1478163

**Published:** 2024-11-14

**Authors:** Sujuan Chen, Mingchen Cai, Guirong Tan, Ruomi Guo, Qiong Liang, Hainan Li, Xiang Liu

**Affiliations:** ^1^ Department of Neurology, The Affiliated Yuebei People’s Hospital of Shantou University Medical College, Shaoguan, Guangdong, China; ^2^ Department of Radiology, The Affiliated Yuebei People’s Hospital of Shantou University Medical College, Shaoguan, Guangdong, China; ^3^ Advanced Neuroimaging Laboratory, The Affiliated Yuebei People's Hospital of Shantou University Medical College, Shaoguan, Guangdong, China; ^4^ Department of Radiology, The Third Affiliated Hospital of Sun Yat-Sen University, Guangzhou, China; ^5^ Department of Pathology, The Third Affiliated Hospital of Sun Yat-Sen University, Guangzhou, China; ^6^ Department of Pathology, Guangdong Sanjiu Brain Hospital, Guangzhou, China

**Keywords:** intravascular large B cell lymphoma, acute demyelinating encephalomyelitis, SARS-CoV-2 reinfection, advanced MR imaging, ChatGPT

## Abstract

The intravascular large B cell lymphoma (IVLBCL) is a rare subtype of lymphoma. The IVBCL is usually found with systemic involvement, with a relative predilection for skin and the central nervous system (CNS), followed by a rapidly progressive course and poor prognosis with a high mortality rate. IVLBCL is difficult to diagnose based on conventional MRI alone. Herein, we presented a previously healthy 59-year-old woman who developed hemiparesis and altered mental status after her reinfection of SARS-CoV-2. The initial MRI revealed non-enhancing lesions in the splenium of the corpus callosum (CC), periventricular, and bilateral subcortical white matter with hyperintensity on diffusion weighted imaging (DWI). The patient was diagnosed with subacute infarction, and she was treated with antithrombotic therapy. Her neurological symptoms continued to deteriorate, and she developed unconsciousness. Her CSF test showed elevated white cell count and positive oligoclonal bands. The follow-up MRI was scanned 16 days later. Compared to the initial MRI, the periventricular and bilateral subcortical lesions enlarged on conventional MRI. The post-contrast 3D black blood Cube images demonstrated multiple parenchymal and diffuse meningeal enhancements and 3D arterial spin labeling showed increased perfusion in the CC splenium. These findings suggested the differential diagnosis of acute demyelinating encephalomyelitis (ADEM) after SARS-CoV-2 reinfection, versus intravascular lymphoma. After the treatment of intravenous immunoglobulin and methylprednisolone, her symptoms significantly improved. The second follow-up MRI two weeks later detected a new unenhanced lesion in the left temporal lobe. A brain biopsy was performed and IVLBCL was diagnosed. We reviewed the brain MRI findings of IVLBCL in the literature with the assistance of ChatGPT. Although less specific, the imaging features including “high signal lesions on DWI, meningeal thickening and enhancement, and masslike lesions” highly suggested the possibility of IVLBCL. The biopsy should be planned after imaging progression. The association between IVLBCL and SARS-CoV-2 reinfection is undefined.

## 1 Introduction

The intravascular large B cell lymphoma (IVLBCL) is a rare subtype of large B-cell lymphoma, characterized by the proliferation of neoplastic lymphoid cells within the lumina of medium and small vessels (1), with a predilection for skin and the central nervous system (CNS). Autopsy data show that 60%-85% of IVLBCL patients have pathological involvement of the nervous system, with more than one-third initially presenting with neurological symptoms (2). The neurological symptoms of IVLBCL are diverse. A meta-analysis of 654 IVLBCL cases reported that over half of the patients had neurological symptoms, with about 82% showing CNS involvement. The most common CNS symptoms were cognitive impairment/dementia (60.9%), paralysis (22.2%), and seizures (13.4%) (3). The clinical course of IVLBCL is aggressive and usually rapidly progressive neurological involvement, the prognosis is usually poor with a high mortality rate. As prompt treatment can prolong the survival of such patients, early diagnosis is critical important. However, especially when the CNS symptoms were the only clinical manifestation, due to the marked variability in clinical presentation and nonspecific laboratory and radiological findings, the diagnosis of cerebral IVLBCL is often difficult.

Herein, we reported consecutive MRI findings, including advanced MRI findings in a biopsy proven IVLBCL case mimicking acute demyelinating encephalomyelitis (ADEM) after SARS-CoV-2 reinfection.

As MRI is recommended as a routine examination for IVLBCL involving CNS, a better understanding of the brain MRI findings of IVLBCL will be crucial for early diagnosis and accurate treatment decisions. We reviewed the literature, with a focus on brain MRI features using the assistance of ChatGPT.

## 2 Case description

**Figure 1:**
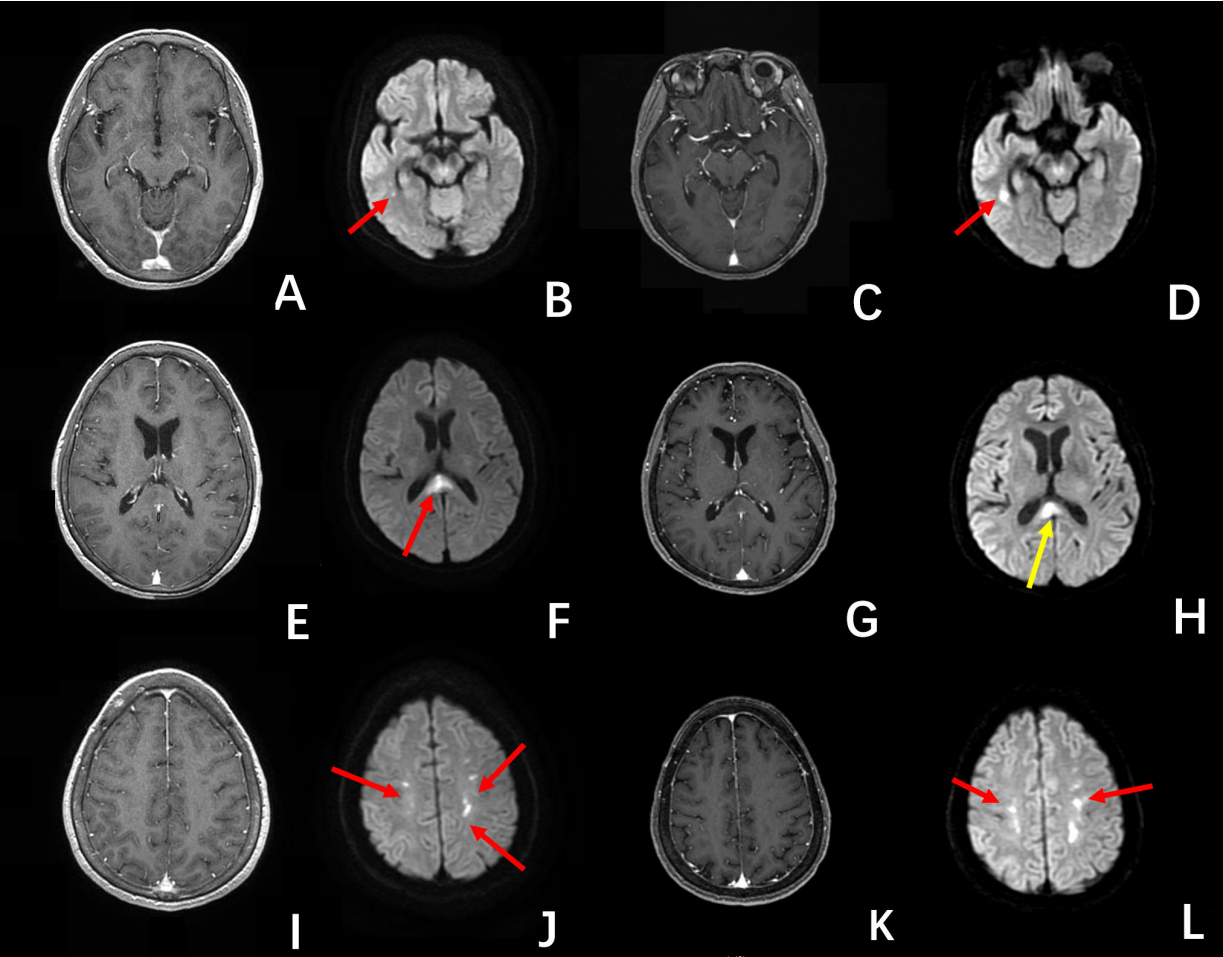
The first MRI **(A, B, E, F, I, J)** showed multiple unenhanced lesions, in the periventricular, splenium of the corpus callosum, and bilateral subcortical regions, with hyperintensity on DWI images (red arrow), were diagnosed as infarctions. The second MRI examination **(C, D, G, H, K, L)** showed except for the smaller lesion in the splenium of the corpus callosum (yellow arrow on **(H)**), the other lesions (red arrow) enlarged. These lesions were un-enhancing on the post-contrast T1-BRAVO images.

A previously healthy 59-year-old woman had first SARS-CoV-2 infection in early January 2023. Her symptoms were unremarkable, without dyspnea and neurological manifestations. She recovered completely within 7 days. In early May 2023, she regained SARS-CoV-2 infection with a mild fever for 3 days. She developed hemiparesis and altered mental status 10 days later, subacute infarction was diagnosed based on scattered lesions in the splenium of the corpus callosum (CC), periventricular and bilateral subcortical white matters with hyperintensity on diffusion weighted imaging (DWI), without contrast-enhancement (**Figures 1A, B, E, F, I, J**).

**Figure 2:**
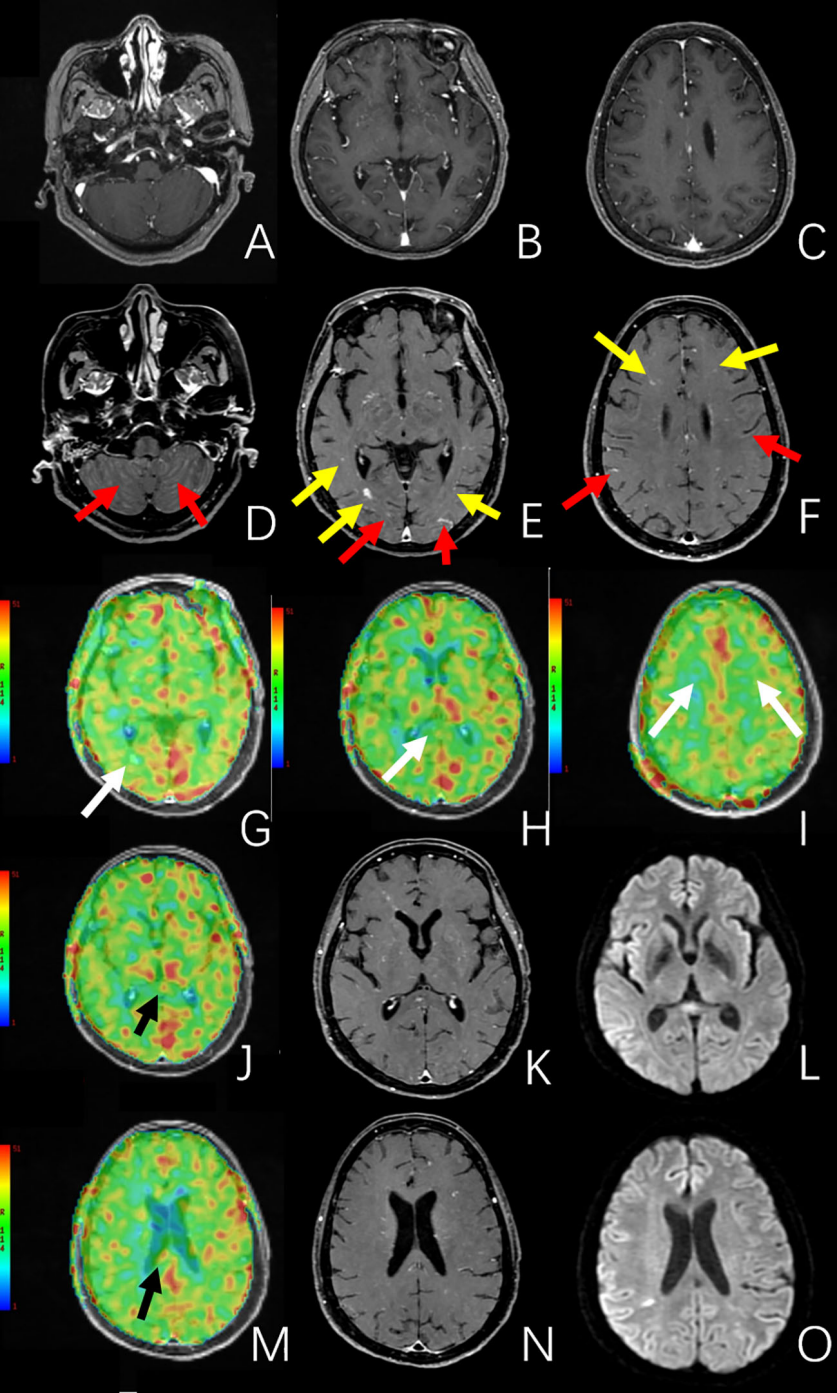
The MRI images of the second MRI examination. **(A–C)** are post-contrast T1-BRAVO images. **(D–F)** are post-contrast 3D black blood Cube images. Compared to the post-contrast T1-BRAVO images, 3D black blood Cube images showed multiple parenchymal (yellow arrow) and meningeal (red arrow) enhancements. **(G–J, M)** are CBF images derived from arterial spin labeling (ASL). **(K, N)** are post-contrast 3D black blood Cube images, and **(L, O)** are DWI images. **(G–I)** showed no increased perfusion in the previously mentioned periventricular, splenium of the corpus callosum, and bilateral subcortical lesions (white arrow). **(J–O)** showed beyond the DWI hyperintensity lesion in the splenium of the corpus callosum, there were multiple unenhanced regions in the splenium of the corpus callosum presenting elevated CBF (black arrow).

Antithrombotic therapy (Bayaspirin 100mg) was admitted. Her neurological symptoms continued to deteriorate, and the unconsciousness ensued. Her cerebral spinal fluid (CSF) test showed elevated white cell count (9×10^6^/L) and positive oligoclonal bands. A repeated MRI after 16 days was obtained on a 3.0 T MR scanner (Discovery 750, GE Healthcare, Milwaukee, WI, USA). DWI was performed using fat-suppressed single-shot spin-echo echo-planar imaging (TR/TE =7,300 ms/77.3 ms, slice thickness = 4.0 mm, slice gap = 0 mm, field of view = 22 ×22 cm, matrix = 130 × 160, NEX = 4) with b = 1,000 s/mm^2^ applied in the x, y, and z directions, and b = 0 s/mm^2^ without motion-probing gradients. This MRI examination showed enlargement of periventricular and bilateral subcortical lesions (**Figures 1C, D, G, H, K, L**). The pseudocontinuous arterial spin labelling (pCASL) was performed using a background suppressed 3D fast spin echo (FSE) technique. The parameters were as follows: TR = 5,337 ms; TE = 10.7 ms; post-labelling delay = 1,525 and 2,525 ms; FOV = 24 × 24 cm; matrix = 512 × 512; slice thickness = 4.0 mm; slice gap = 0 mm; NEX = 3. The parameters of the post-contrast T1-weighted brain volume imaging (T1-BRAVO) included TR = 8.47 ms, TE = 3.25 ms, TI = 450 ms, slice thickness = 1 mm, flip angle = 15°, matrix = 256 × 256 mm, FOV = 256 mm, bandwidth = 31.25 Hz, number of averages = 1. The post-contrast axial 3D T1-weighted black blood Cube was acquired using the following parameters (TR = 800 ms, TE = 16.32 ms, slice thickness = 0.8 mm, echo-train length = 40, matrix = 256 × 256 mm, FOV = 240 mm, number of averages = 2). This MRI examination showed enlargement of periventricular and bilateral subcortical lesions (**Figures 1C, D, G, H, K, L**). These lesions were unenhanced on the post-contrast T1-BRAVO images, but multiple parenchymal and diffuse meningeal enhancements were detected on post-contrast 3D black blood Cube images (4), **Figures 2A–F**. The susceptibility weighted imaging (SWI) showed a small hypointensity lesion (not shown) in the right frontal lobe which was remote from the right frontal lesions with DWI hyperintensity. The MR dynamic susceptibility contrast (DSC) perfusion weighted imaging (PWI) findings were unremarkable (not shown). The DWI hyperintensity lesions in the splenium of CC, periventricular, and bilateral subcortical white matter didn’t present increased cerebral blood flow (CBF) (**Figures 2G–I**), which may be consistent with the demyelinating and inflammatory process. However, patchy unenhanced regions within the rest of the splenium showed elevated CBF (**Figures 2J–O**), which indicated potential cancer characteristics.

These findings suggested the differential diagnosis between ADEM after SARS-CoV-2 reinfection, and intravascular lymphoma. In combination of MRI findings, clinical presentations and laboratory results, the clinical diagnosis of “demyelinating disease” was decided.

**Figure 3:**
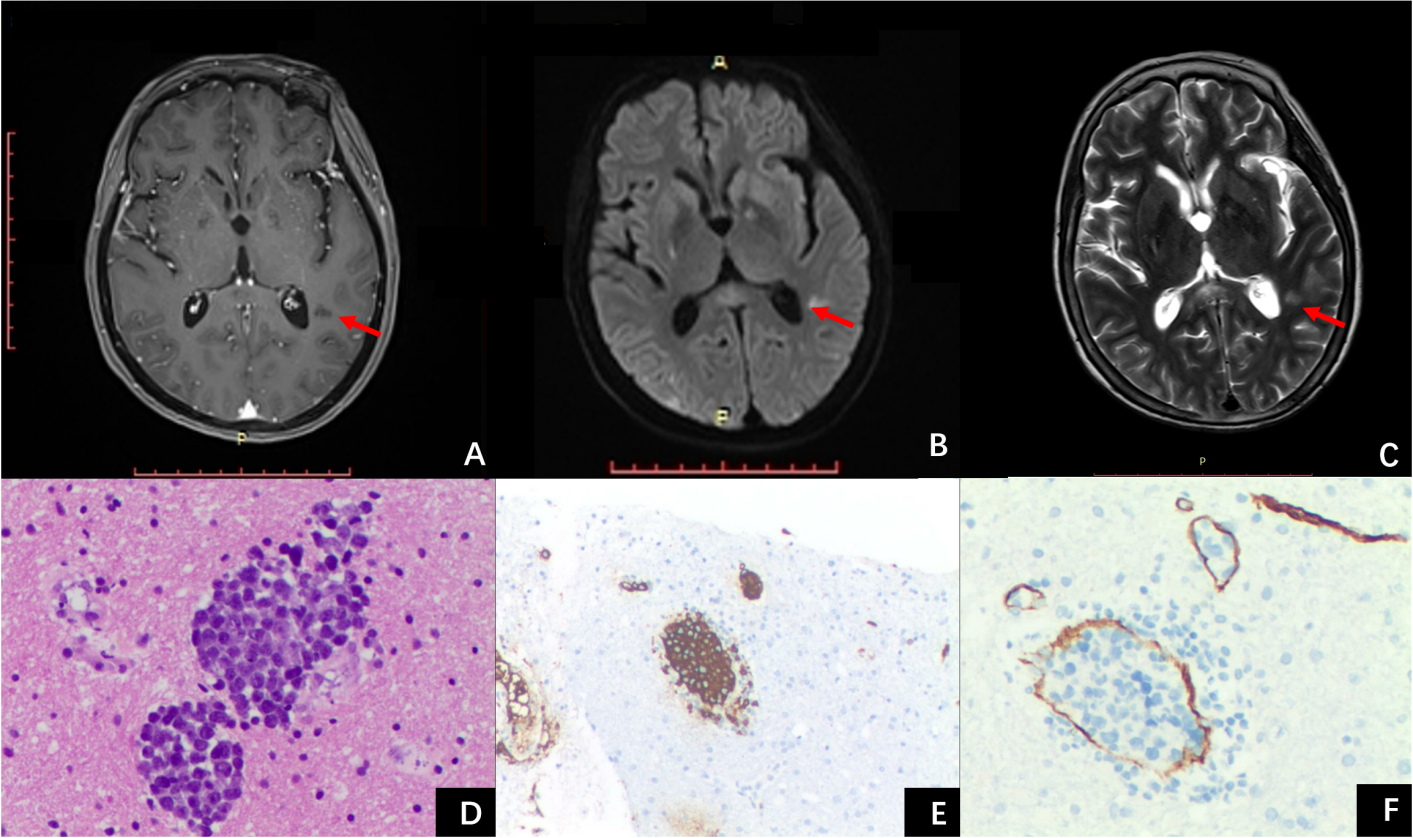
The third MRI examination and histopathology images. The post-contrast T1 MPRAGE image **(A)** showed a new un-enhanced lesion in the right temporal lobe, with hyperintensity on DWI **(B)** and T2WI **(C)**. **(D)** hematoxylin-eosin, ×20, shows the accumulation of tumor cells within the vascular space, and the atypical cells with medium-sized to large round, slightly irregular, nuclei and distinct nucleoli. **(E)** CD20 immunohistochemistry highlights the neoplastic cells. **(F)** CD34 immunohistochemistry highlights the vessels.

She was treated with a combination therapy of intravenous immunoglobulin (0.4 g/kg) and methylprednisolone (methylprednisolone, 1g three day, 500mg three days, reduced every three days), and her symptoms significantly improved. Two weeks later, the follow-up MRI revealed a new unenhanced lesion in the left temporal lobe (**Figures 3A–C**), a biopsy was performed, and intravascular large B cell lymphoma (IVLBCL) was diagnosed (**Figures 3D–F**). She received the treatment of Rituximab, Temozolomide and Orelabrutinib. The patient showed neurological recovery and her MRI at 6-month follow-up after biopsy showed stable cerebral lesions.

## 3 Discussion

We reported a pathology confirmed cerebral IVLBCL with conventional MRI findings mimicked subacute stroke and demyelinating disease. The post-contrast 3D black blood Cube revealed more “occult” parenchymal and diffuse meningeal enhancing lesions. The arterial spin labeling (ASL) detected abnormal patchy increased perfusion in the splenium of CC indicating the possibility of IVLBCL. The appearance of enlarged and new lesions after antithrombotic therapy, intravenous immunoglobulin, and methylprednisolone treatments, emphasized the importance of biopsy for early diagnosis of IVLBCL.

The largest lesion of the present case is located in the splenium of CC, and this patient presented with diffuse parenchymal and meningeal enhancement. The imaging features including lesion location and enhancement pattern, as well as the clinical history of SARS-CoV-2 reinfection and deteriorated neurological symptom, were similar to a SARS-CoV-2-related ADEM case which was recently published in Neurology (5). In addition, the CSF test result of positive oligoclonal bands is usually interpretated as the indicator of “demyelinating disease”. This case showed the complexity of MRI findings of cerebral IVLBCL lesions, and the difficulty of early accurate diagnosis of IVLBCL. For a better understanding of MRI characteristics of IVLBCL, we reviewed the recent literature, focusing on the published MRI findings of cerebral IVLBCL lesions with the assistance of ChatGPT.

Previous IVLBCL studies are limited in the review of MRI findings of cerebral IVLBCL lesions. To the best of our knowledge, our literature review is the first review focusing on the MRI features of such cerebral IVLBCL lesions. We reviewed 70 papers between 2005 and 2024, including 134 pathology-confirmed IVLBCL cases who underwent brain MRI examinations, **Supplementary Table S1**.

Yamamoto et al. (6) first categorized five types of MRI findings of IVLBCL involving the brain as 1) infarctlike lesions, 2) nonspecific white matter lesions, 3) meningeal enhancement, 4) masslike lesions, and 5) hyperintense lesions in the pons on T2-weighted imaging (T2WI). In the study of Abe et al. (7), they suggested four-types classification as 1) hyperintense lesion in the pons on T2WI, 2) nonspecific white matter lesions, 3) infarctlike lesions, and 4) meningeal thickening and/or enhancement. Both classification systems are subjective, with a mixture of imaging signal abnormalities and disease entities, thus, their utilizations in clinical practices were limited.

We discussed the MRI findings of the collected studies in the following sections “Enhancement degrees and patterns”, “DWI”, and advanced MRI techniques of “MR PWI” and “SWI”, and dynamic MRI changes.

### 3.1 Enhancement degrees and patterns

Among 134 collected cases, only 59 (44%) had post-contrast MRI scans. Approximately 76.3% (45/59) of them presented enhancing lesions. This indicates that tumor enhancement is an imaging characteristic in cerebral IVLBCL lesions, which is similar to the primary central nervous system lymphoma (PCNSL).

The enhancement patterns of cerebral IVLBCL lesions could be categorized into meningeal enhancement and parenchymal enhancement. The meningeal enhancements were observed in 17 (17/45) cases. The parenchymal enhancements in the brain were detected in 32 (32/45) cases. These 4 cases (4/45) presented both meningeal enhancement and parenchymal enhancement.

The enhancement patterns in the present case are generally consistent with previous studies. It was very interesting that we found the post-contrast 3D black blood Cube not only improved the visibility of the lesions detected on conventional post-contrast T1-BRAVO images but also could reveal additional parenchymal lesions and diffuse meningeal enhancements that were not visualized on conventional post-contrast T1-BRAVO images. Our results were similar to the previous study by Vandermeersch et al. (8). The imaging advantage of post-contrast 3D black blood Cube compared to conventional post-contrast T1-BRAVO needs to be identified in future studies with large cohorts. This also raises suspicion of the possibility of the underestimation of meningeal enhancement in cerebral IVLBCL lesions in the previous studies due to the technical limitation of conventional post-contrast T1-BRAVO.

### 3.2 DWI

Previous studies have shown that PCNSL often exhibits diffusion restriction on imaging (9–11), with apparent diffusion coefficient (ADC) values around 1.1 × 10^−3^ mm²/s, which helps differentiate PCNSL from other brain lesions (12) While IVLBCL, a subtype of lymphoma, has been reported in many studies to show high signals on DWI sequences, detailed descriptions of diffusion restriction in IVLBCL are scarce. Among the 134 cases reported in the collected literature, 63 showed hyperintensities on DWI, 19 were reported as diffusion restriction, 17 were described as low ADC signals (one case only described the ADC map without providing DWI signals (13)), 3 cases showed partial ADC reduction or diffusion restriction within the lesion, and 2 cases explicitly stated no ADC signal reduction. This suggests varying interpretations of “high signal on DWI sequence” in published IVLBCL studies among authors, and it remains unclear whether IVLBCL exhibits diffusion restriction or if DWI high signal is specific for diagnosing IVLBCL. Zhao et al. (14) and Kageyama et al. (15) suggested that observing dynamic changes in DWI and ADC signals, such as persistent diffusion restriction, could support the diagnosis of IVLBCL and help to distinguish IVLBCL from cerebral infarction. These conclusions need to be identified by future studies. In addition, the diagnostic values of DWI and ADC in differentiating between IVLBCL and demyelinating diseases are unclear.

In our present case, there were no restricted diffusions in the MRI examinations, and the follow-up MR after anticoagulant therapy showed an increase of lesions with hyperintensity on DWI. These findings were useful for the differential diagnosis of ischemic stroke. These findings indicated that DWI high signal alone is not a specific feature for the diagnosis of cerebral IVLBCL.

### 3.3 MR PWI

There were only two published studies that described imaging findings of MR DSC-PWI in cerebral IVLBCL lesions. Carvalho RM (16) reported a case of IVLBCL with multiple enhancing lesions in the brain parenchyma, without significant perfusion abnormalities on cerebral blood volume (CBV) maps. A Pons-Escoda (10) noted that when IVLBCL invades extravascularly, enhancing tumor islands may form in the brain parenchyma, and can exhibit a low to moderate increase of CBV.

In our case, the findings of MR DSC-PWI were similar to previous studies (10, 16). Our report is the first study describing ASL findings in cerebral IVLBCL. The CBF abnormalities suggested the possibility of early involvement of brain tissue by IVLBCL. Its diagnostic value needs to be identified in future studies.

### 3.4 SWI

SWI is an MRI sequence that is highly sensitive to hemorrhage and thus has been used to detect IVLBCL lesions with bleeding (17, 18). The previous 8 studies with SWI applied in IVLBCL included 17 patients. Microbleeds or hemorrhages associated with cerebral IVLBCL lesions can appear as hyposignal on SWI sequences (18). Most lesions are located within areas of high intensity on T2WI (18). Additionally, some hemorrhages may present as high signal on T1WI (19, 20). The mechanism of hemorrhage in IVLBCL may involve tumor cell invasion of the vascular wall (21, 22), leading to microvascular rupture, while hematomas may result from the rupture of medium-sized arteries (18). Pons-Escoda A (10) reported that the presence of hemorrhage in dynamically changing infarct-like or progressively masslike lesions on T2WI and DWI should raise suspicion for IVLBCL. For early detection of IVLBCL, lesions may initially appear as low signals on SWI, with no significant abnormalities on other conventional sequences. As the disease progresses, T2 fluid attenuated inversion recovery (T2 FLAIR) hyperintense lesions may develop in the corresponding regions (18).

In the present case, an isolated hypointensity lesion was detected on SWI, without evidence of imaging abnormalities on other sequences. It kept stable in the follow-up MR examinations. The mechanism of this lesion is still unclear. It may be associated with the early detection of complex and variable IVLBCL lesions involving the brain.

### 3.5 Dynamic MRI changes

After a review of the literature, we found information on initial diagnoses within 53 cases. The majority (42 cases, 79.2%) of these initial diagnoses were stroke or demyelinating disease or vasculitis, including 28 of stroke, and 14 as demyelination or vasculitis. These findings demonstrated that the initial diagnoses of demyelinating disease and vasculitis were common in the true world of clinical management of cerebral IVLBCL, which was not included in the classification systems by Yamamoto (6) and Abe (7).

Biopsy is the only way to obtain a definite diagnosis of IVLBCL. A rapid decision of brain biopsy is recommended based on early suspicion of cerebral IVLBCL. In the previous studies, 33 cases had follow-up MRI examinations before brain biopsies or autopsies. Among them, 31 cases presented enlarged or new cerebral lesions, suggesting tumor progression. Therefore, imaging progressive worsening despite experimental treatments highly suggests that IVLBCL should be considered in the differential diagnosis and subsequently, a biopsy should be planned for the early diagnosis.

In the present case, a new lesion appeared following antithrombotic therapy and treatment of methylprednisolone and immunoglobulin. Subsequent biopsy confirmed IVLBCL. The diagnostic experience of this case supported that the evaluation of the dynamic MR changes was a key component of clinical workflow for the prompt decision-making of brain biopsy in such IVLBCL patients.

## 4 Literature review with assistance of ChatGPT

With the rapid development of artificial intelligence (AI) technology in the medical field, multiple studies have demonstrated the applicability of the deep learning natural language processing model, Generative Pre-trained Transformer (GPT), in radiological diagnosis. These applications mainly include disease diagnosis assistance (23) and interpretation of radiology reports (24). However, there are few reports regarding to the application of ChatGPT in the radiological diagnosis of rare diseases. The 11 papers including detailed descriptions of MRI findings of 57 patients (**Table 1**) were selected by two radiologists from the collected literature. The MRI information was inputted into ChatGPT-4o, which determined whether there were specific features of cerebral IVLBCL after learning (**Supplementary Materials**). In addition, we also reviewed the literature for the evidence of oligoclonal bands in cerebral IVLBCL lesions.

**Table 1:** Summary of major MRI findings of cerebral IVLBCL lesions in the literature.

References	Number of cases	MR			
		DWI	T1WI/T2WI/FLAIR	SWI	Post-contrast MRI
Yamamoto et al. Characteristics of intravascular large B-cell lymphoma on cerebral MR imaging. AJNR Am J Neuroradiol. 2012	Case #: 11 Male: 4 Female: 7 Age: 63-84Y	2 cases: hyperintense areas on T2WI with diffusion restriction (infarctlike lesions).	2 cases: poorly margined hyperintense lesions on T2WI without mass effect or abnormal enhancement (nonspecific white matter lesions). 5 cases: hyperintense lesions in the pons on T2WI.	N/A	2 cases: abnormal enhancement along the surface of the cortex with a pia-arachnoid pattern extending on postcontrast T1WI (meningeal enhancement). 1 case: multiple intraparenchymal focal enhanced lesions and mass effect (masslike lesions).
Abe et al. Clinical value of abnormal findings on brain magnetic resonance imaging in patients with intravascular large B-cell lymphoma. Ann Hematol. 2018	Case#: 33 Male: 17 Female: 16 Median Age: 73.2 years (IQR 65.8–76.4)	8 cases: hyperintense areas on T2WI with diffusion restriction.	14 cases: poorly margined hyperintense lesions on T2WI with or with- out mass effect and abnormal enhancement. 19 cases: hyperintense lesions in the pons on T2WI.	N/A	4 cases: Meningeal enhancement.
Hung et al. Brain biopsy-proven intravascular lymphomatosis presenting as rapidly recurrent strokes-two case reports. Acta Neurol Taiwan. 2014	Case#: 2 Case 1: 70Y female	1^st^ MRI: DWI showed lesions in the left parieto-occipital region, which were hyperintense on diffusion weighted imaging (DWI) and hypointense on apparent diffusion coefficient (ADC) maps. 2^nd^ MRI: DWI showed multiple bilateral ischemic lesions, of which some were hyperintense on DWI and hypointense on ADC maps, with minimal hemorrhagic transformation. 3^rd^ MRI: showed multiple multistage infarctions, minimal hemorrhage in bilateral cerebral hemispheres.	1^st^ MRI: FLAIR showed hyperintense lesions in the left parieto-occipital region. 2^nd^ MRI: FLAIR showed bilateral hyperintense lesions, 3^rd^ MRI: FLAIR showed bilateral hyperintense lesions,	N/A	N/A
	Case 2: 65Y female	1^st^ MRI: DWI showed a right frontal infarct, which was hyperintense on DWI and showed restricted diffusion on ADC maps. 2^nd^ MRI: DWI showed there were new hyperintense lesions on DWI in bilateral cerebral hemispheres. 3^rd^ MRI: DWI showed multiple hyperintense lesions on DWI in bilateral cerebral hemispheres. These lesions showed restricted diffusion on ADC maps.	1^st^ MRI: FLAIR showed hyperintense lesions in the right frontal, with new lesions. 2^nd^ MRI: FLAIR showed progression of lesion size in the right frontal area, with new lesions.	N/A	No enhancement was observed.
Jin et al. Clinical features and imaging manifestations for intravascular large B-cell lymphoma. Zhong Nan Da Xue Xue Bao Yi Xue Ban. 2023	51y female	DWI showed the lesion in the left occipital lobe, with few high signals at the edges on DWI and low values of the corresponding ADC.	Multiple long T1 and short T2 signal foci in the bilateral frontal-parietal-occipital lobe, right temporal lobe, and bilateral cerebellar hemispheres with visible occupancy effects, as well as a localized piece of short T1 signal in the left occipital lobe lesion.	SWI showed multiple microhemorrhagic foci.	Meningeal enhancement.
Turin et al. Central nervous system intravascular lymphoma leading to rapidly progressive dementia. Proc (Bayl Univ Med Cent). 2021	52Y male	DWI showed multifocal areas of punctate diffusion restriction in the bilateral subcortical white matter.	N/A	N/A	Post-contrast T1WI showed diffuse leptomeningeal enhancement and faint contrast enhancement in the multifocal areas in the bilateral subcortical white matter as punctate hyperintensities
Wu et al. Intravascular large B-cell lymphoma presenting as rapidly progressive dementia and stroke: A case report. Medicine (Baltimore). 2021	47Y female	1^st^ MRI: DWI showed bilateral dotted hyperintense lesion in periventricular white matter, centrum semiovale, and corpus callosum. 2^nd^ MRI: the lesions decreased significantly after receiving pulsed steroid therapy. 3^rd^ MRI: DWI showed the lesions were enlarged and increase.	1^st^ MRI: FLAIR showed bilateral multiple hyperintense lesions in periventricular white matter, centrum semiovale, corpus callosum, and cerebellum. 2^nd^ MRI: the lesions decreased significantly after receiving pulsed steroid therapy. 3^rd^ MRI: FLAIR showed the lesions were enlarged and increase.	N/A	1^st^ MRI: light enhancement in periventricular white matter. 2^nd^ MRI: the lesions decreased significantly after receiving pulsed steroid therapy. 3^rd^ MRI: some lesions had open-ring enhancement.
Miyake et al. Intravascular large B-cell lymphoma presenting with hearing loss and dizziness: A case report. Medicine.2019.	66Y male	1^st^ MRI: DWI showed multiple high-intensity lesions in bilateral cerebral white matter and cortex, posterior limbs of the internal capsule, and cerebellar hemispheres. Reduction in signals at those lesions was seen on ADC maps.	1^st^ MRI: T2WI/FLAIR showed multiple hyperintense lesions bilaterally in the cerebral white matter and basal ganglia.	N/A	2^nd^ MRI: Post-contrast T1WI showed contrast-enhancing high-signal areas along the cortex in regions of the right frontal lobe, bilateral parietal lobe, and left parieto-occipital region.
Rota et al. Intravascular large B-cell lymphoma: a forgotten stroke “mimic”. Acta Neurol Belg.2020	62Y male	1^st^ MRI: DWI negative.	1^st^ MRI: T2WI showed hyperintense subcortical lesions in the frontal and temporal regions. 2^nd^ MRI: T2WI lesions increased despite steroid treatment.	N/A	2^nd^ MRI: Post-contrast T1WI showed the frontal cortico-subcortical lesion with gadolinium enhanced.
Vandermeersch et al. Intravascular large cell B lymphoma presenting as central nervous system pseudo-vasculitis: A rare diagnostic challenge. Neuroradiol J. 2023	50Y female	1^st^ MRI: DWI showed left postcentral gyrus with corresponding areas of cortico-subcortical hemorrhagic lesions.	1^st^ MRI: multiple tumefactive intraparenchymal lesions characterized by a hypersignal on T2WI and T2-FLAIR; an ill-defined T2-WI hyperintense lesion in the central pons. 2^nd^ MRI: FLAIR showed new foci of hyperintensity and significative progression of the pre- and post-central tumefactive lesions.	1^st^ MRI:T2*-weighted image showed left postcentral gyrus with corresponding areas of cortico-subcortical hemorrhagic lesions. 2^nd^ MRI: T2*-weighted image showed significative progression of the cortical subcortical petechial foci.	1^st^ MRI: Post-contrast T1WI showed no enhancement. 2^nd^ MRI: 3D-T1 (SPGR) sequence showed no enhancement; post-contrast black-blood images showed subtle signs of blood-brain-barrier rupture.
Sharma et al. Intravascular T-cell lymphoma: A rare, poorly characterized entity with cytotoxic phenotype. Neuropathology. 2017	62Y female	1^st^ MRI: DWI showed a small hyperintense lesion was detected in the right cerebellum. 2^nd^ MRI: near complete resolution of the above lesions. 3^rd^ MRI: multiple areas of restricted diffusion in the right lateral splenium, left corona radiata, right parietal convexity and left superior vermis.	3^rd^ MRI: T2FLAIR revealed abnormal high signal areas in the left frontal cingulate gyrus, left occipital cortex, and right splenium, as well as multiple areas of vasogenic edema.	N/A	1^st^ MRI: Two enhanced lesions in the left frontal cingulate gyrus and right parietal cortex. 2^nd^: Lesions disappeared. 3^rd^: Post-contrast T1WI showed gyriform enhancement of left frontal operculum and right frontal cortex.
Xu et al. Multiple white matter lesions combined with subcortical microbleeds in patients with intravascular large B-cell lymphoma. Quant Imaging Med Surg. 2023	Case#: 4 Case 1: 56Y male	DWI showed periventricular white matter, frontal lobe, parietal lobe, splenium of corpus callosum, basal ganglia, left cerebellum, and left cerebral peduncle lesions were partially hyperintense, but the corresponding ADC signal was not reduced.	diffuse hypointensity on T1WI, hyperintensity on T2WI and T2-FLAIR in the periventricular white matter, frontal lobe, parietal lobe, splenium of corpus callosum, basal ganglia, left cerebellum, and left cerebral peduncle.	SWI showed multiple punctate to small clusters of microbleeds in the subcortical areas of the bilateral cerebral hemispheres, cerebellum, basal ganglia, and thalamus.	no significant abnormal enhancement.
	Case 2: 60Y male	DWI showed multiple hyperintense lesions in the bilateral frontal lobe, parietal lobe, occipital lobe, left temporal lobe, splenium of corpus callosum, and bilateral cerebellar hemisphere. These lesions showed reduced signal intensity on the corresponding ADC.	multiple hypointense lesions on T1WI, and hyperintense lesions on T2WI and T2-FLAIR in the bilateral frontal lobe, parietal lobe, occipital lobe, left temporal lobe, splenium of corpus callosum, and bilateral cerebellar hemispheres.	N/A	Post-contrast T1WI showed patchy abnormal enhancement in the right frontal lobe, as well as thickening and linear enhancement of the right meningeal and subcortical gyrus-like enhancement.
	Case 3: 67 Y male	1^st^ MRI revealed multiple subacute cerebral infarctions (details of sequences are unclear). 2^nd^ MRI (After stenting): DWI and ADC maps showed partial diffusion restriction in bilateral frontal lobe, parietal lobe, occipital lobe, temporal lobe, corpus callosum, periventricular white matter, and left basal ganglia lesions.	2^nd^ MRI (After stenting) showed multiple hypointense lesions on T1WI, hyperintense lesions on T2WI, and T2-FLAIR in the bilateral frontal lobe, parietal lobe, occipital lobe, temporal lobe, corpus callosum, periventricular white matter, and left basal ganglia.	SWI showed numerous punctate to small clusters of microbleeds in the subcortical areas of the bilateral cerebral hemispheres.	2^nd^ MRI (After stenting): abnormal enhancement of periventricular white matter.
	Case 4: 31Y female	some lesions didn’t show hyperintensity on DWI	T1WI showed a large area of hyperintensity. T2WI/FLAIR showed a corresponding hypointensity, surrounded by patchy edema in the bilateral cerebral hemispheres and right cerebellar hemisphere.	SWI demonstrated numerous microbleeds and superficial siderosis in the cortical and subcortical white matter of the bilateral cerebral hemispheres.	Post-contrast T1WI showed diffuse abnormal enhancement of the brain parenchyma.

N/A, Not Applicable.T2* relaxation refers to the decay of transverse magnetization (25).

In brief, after learning of MRI findings of reported cerebral IVLBCL cases, ChatGPT concluded that the common imaging findings including “High signal lesions on DWI, T2/T2 FLAIR Hyperintensities, Hemorrhagic Lesions, meningeal thickening and enhancement, Pontine Involvement and Masslike lesions” had high recognition value in clinical practice, but less specific for accurate diagnosis.

ChatGPT only found one study that showed the presence of identical oligoclonal bands in the CSF of a patient with IVLBCL presenting as longitudinally extensive myelitis (26). To the best of our knowledge, this is the first report regarding the elevated oligoclonal bands in a patient who initially presented with cerebral IVLBCL. This review may suggest the possible association between oligoclonal bands in the CSF and IVLBCL, and it also indicates the importance of careful interpretation of the oligoclonal bands in such patients with IVLBCL mimicking ADEM.

The conclusions of ChatGPT are aligned with those of human experts. The major reasons for ChatGPT-4o can’t summarize specific MRI features for accurate diagnosis of cerebral IVLBCL may be related to the small amount of published MRI studies due to the rarity of this tumor, and limited information on MRI findings in these published studies. Overall, our preliminary results demonstrate that ChatGPT possesses a certain level of imaging diagnostic reasoning and has great potential in the radiological diagnosis of rare diseases.

## 5 Conclusion

We reported MRI findings of a case with pathology-confirmed cerebral IVLBCL mimicking ADEM after SARS-CoV-2 reinfection on conventional MRI. To the best of our knowledge, this is the first case report showing IVLBCL and SARS-CoV-2 infection. The association between IVLBCL and SARS-CoV-2 reinfection is undefined. Assisted with ChatGPT, we reviewed MRI findings of published cerebral IVLBCL cases. Although there are no definite imaging features for the diagnosis, the imaging features of “high signal lesions on DWI, meningeal thickening and enhancement, and masslike lesions” highly suggested the possibility of IVLBCL. The advanced MRI techniques can reveal more “occult” parenchymal and diffuse meningeal enhancing lesions, and their molecular and metabolism changes. The biopsy should be planned after imaging progression following experimental treatments. Using ChatGPT to assist in diagnosing such rare cerebral tumors in the future could become a new method for improving the accuracy and efficiency of diagnosing rare diseases.

## Data Availability

The original contributions presented in the study are included in the article/**Supplementary Material**. Further inquiries can be directed to the corresponding author.
