# Supplementary material for: Case report: Intravascular large B cell lymphoma mimicking acute demyelinating encephalomyelitis after SARS-CoV-2 reinfection: diagnostic value of advanced MRI techniques and the literature review with the assistance of ChatGPT
Source: Front Immunol. 2024 Nov 14;15:1478163. doi: 10.3389/fimmu.2024.1478163 (PMC11602322; doi:10.3389/fimmu.2024.1478163)
Supplement: Supplementary file 1 [file DataSheet1.docx]

**Supplementary document ：ChatGPT Literature Analysis and Summary Workflow**

**Section 1：**

This study selected 11 articles from the collected literature, extracting imaging information. These articles contain comprehensive MR imaging descriptions and clear diagnostic processes, intended for learning by ChatGPT-4o.

1. The prompt: Hello, ChatGPT, I am a radiologist studying the MR features of intravascular lymphoma. Based on preliminary literature screening, I have selected the following 11 articles as study objects and extracted the imaging findings. Please learn from these contents. I will send the study content to you in multiple parts. After each part, please reply with "Learned." Do you understand?

2) Upload all the documents to ChatGPT-4o for learning, then ask it three questions and record the initial responses：

**Question1 ：**Analyze these cases and answer whether intravascular lymphoma has common MR imaging features？


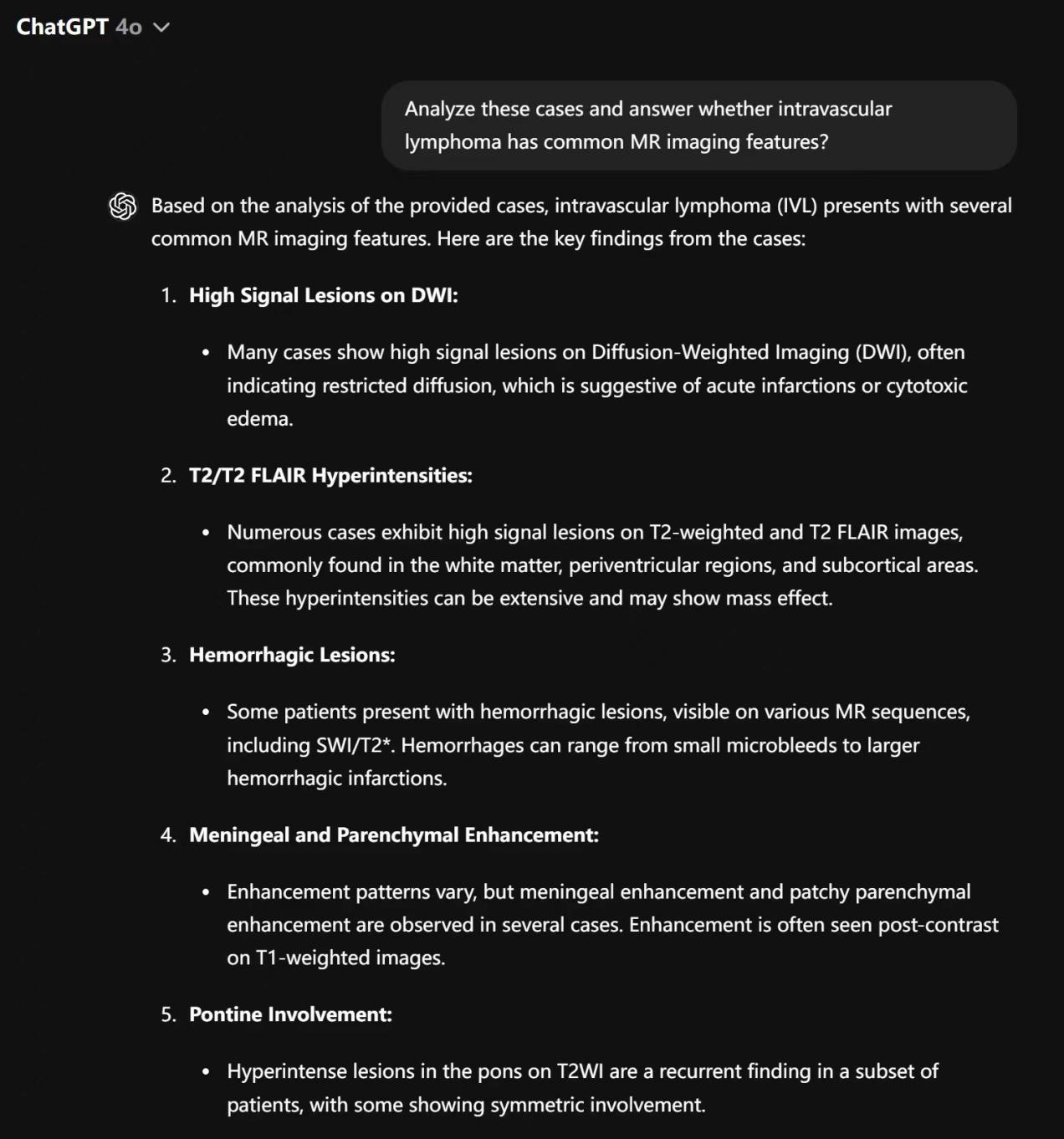


Figure1 ：Queried ChatGPT-4o on whether intracranial lymphoblastic lymphoma has common

imaging features on MRI.

**Question 2 ：**Do these MR imaging features have specificity?


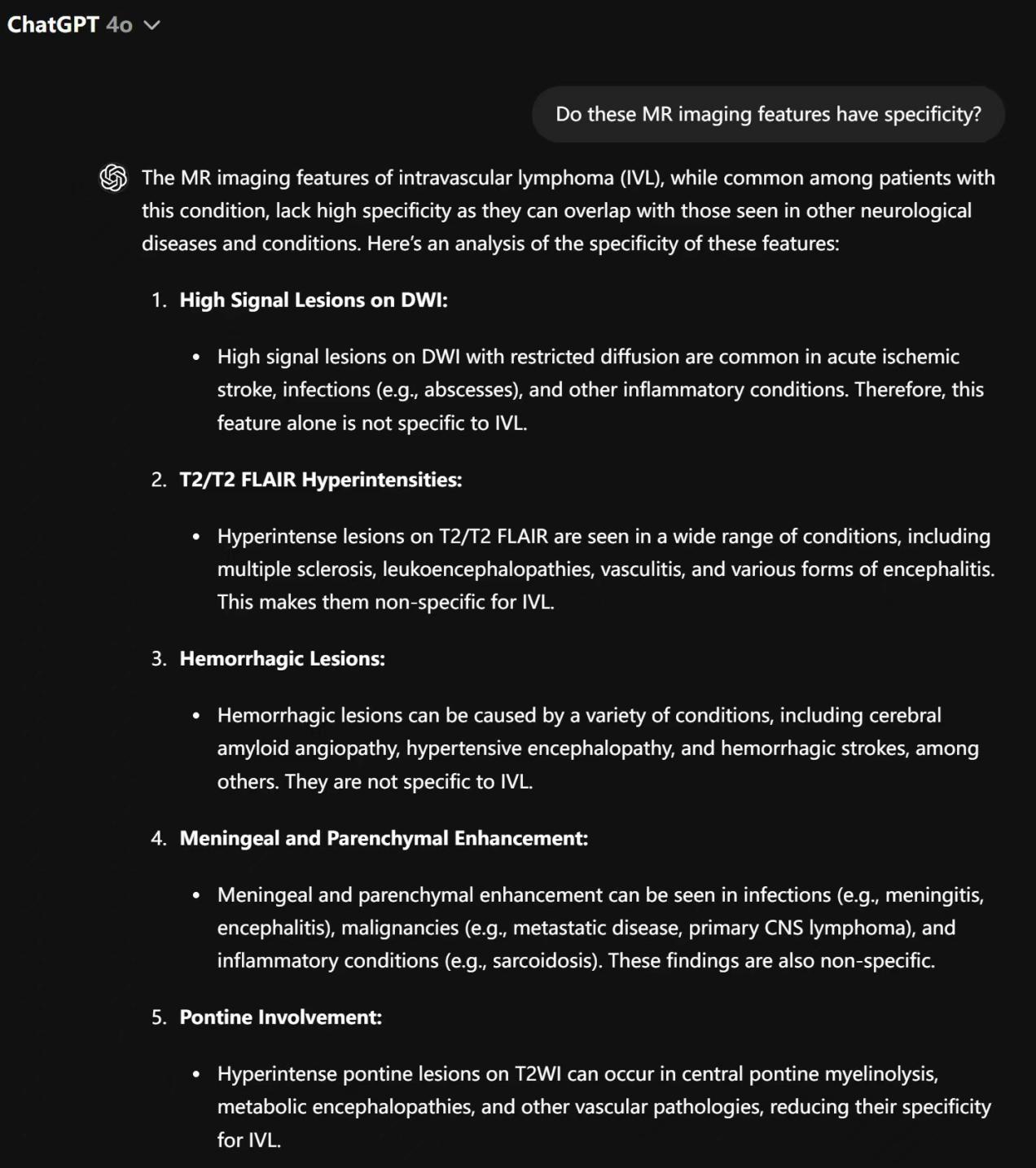


Figure 2: Queried ChatGPT-4o on MR imaging features have specificity.

**Question 3 ：**Can these MR imaging features exclude stroke, demyelination, and vasculitis？


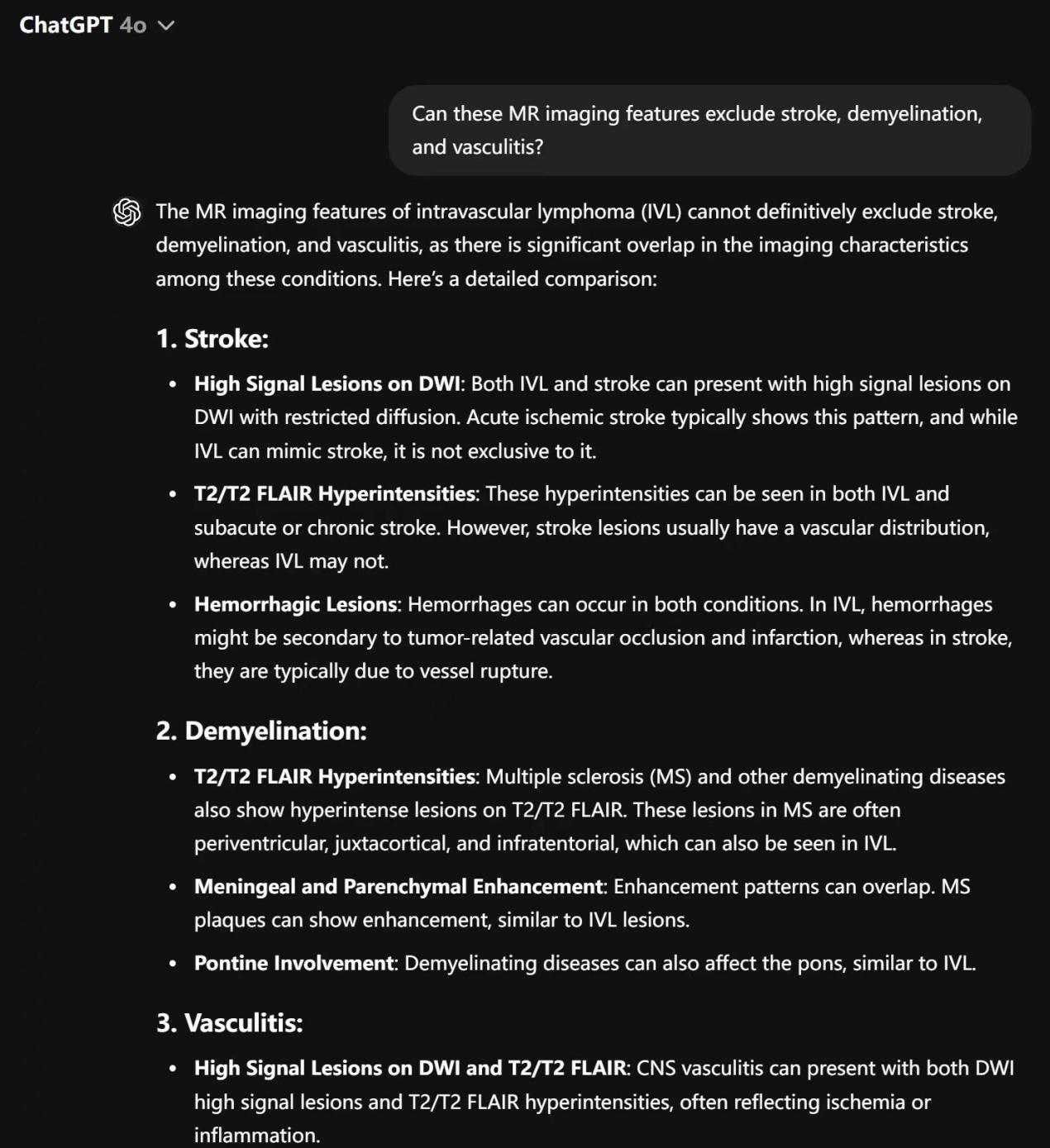


Figure 3: Queried ChatGPT-4o on MR imaging features exclude stroke, demyelination, and vasculitis.

**Section 2：**

Utilize ChatGPT-4o to search for literature regarding the presence of oligoclonal bands in cerebral IVLBCL lesions.

**The prompt:**  Are there studies related to positive oligoclonal bands in IVL? Please provide specific references.


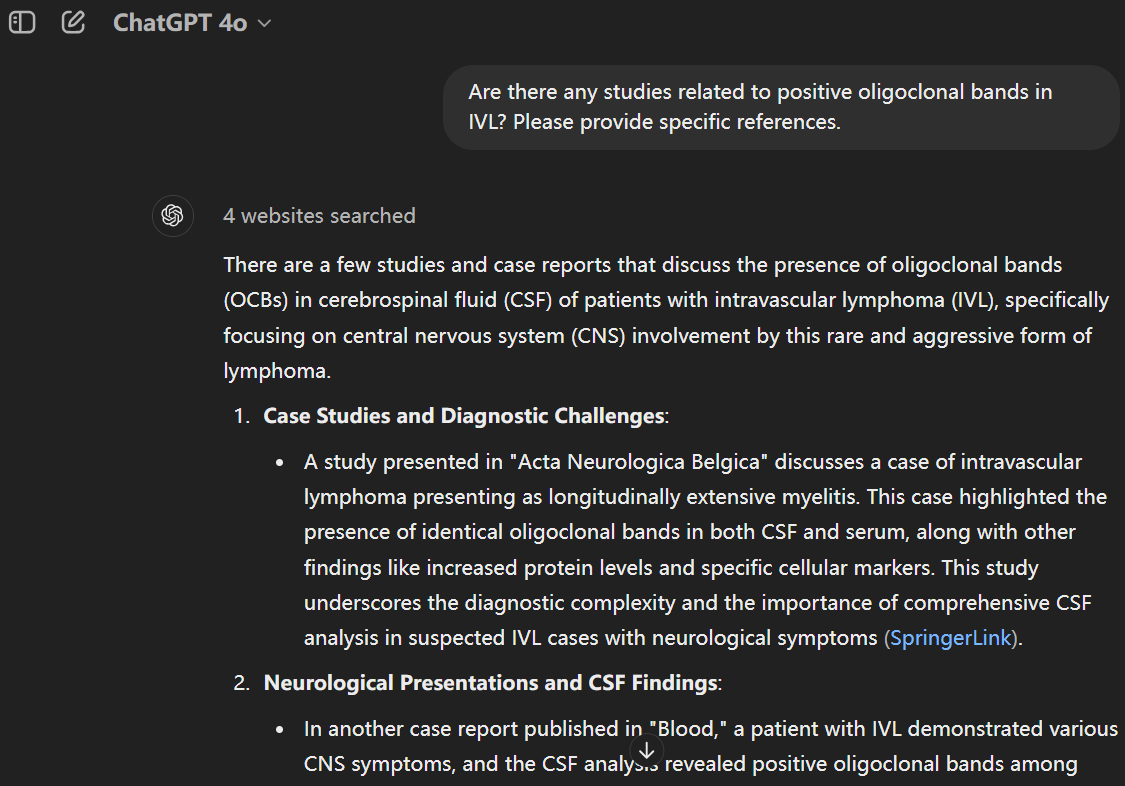


Figure 4：Queried ChatGPT-4o for literature regarding the presence of oligoclonal bands in cerebral IVLBCL lesions.
